# A randomized controlled trial comparing PF-06438179/GP1111 (an infliximab biosimilar) and infliximab reference product for treatment of moderate to severe active rheumatoid arthritis despite methotrexate therapy

**DOI:** 10.1186/s13075-018-1646-4

**Published:** 2018-07-27

**Authors:** Stanley B. Cohen, Rieke Alten, Hideto Kameda, Tomas Hala, Sebastiao C. Radominski, Muhammad I. Rehman, Ramesh Palaparthy, Karl Schumacher, Susanne Schmitt, Steven Y. Hua, Claudia Ianos, K. Lea Sewell

**Affiliations:** 1 Metroplex Clinical Research Center, 8144 Walnut Hill Lane, Suite 810, Dallas, TX 75231 USA; 2 Schlosspark-Klinik University Medicine, Heubnerweg 2, 14059 Berlin, Germany; 3 Toho University Ohashi Medical Center, 2-17-6, Ohashi Muguro-ku, Tokyo, 153-8515 Japan; 4 Center for Clinical and Basic Research, Trida Miru 2800, 530 02 Pardubice, Czech Republic; 5 Universidade Federal do Paraná, Rua General Carneiro, 181 - Alto da Glória, Curitiba, PR 80.060-900 Brazil; 6 Pfizer Inc., 1 Burtt Road, Andover, MA 01810 USA; 7 Pfizer Inc., 10777 Science Center Drive, CB1/2103, San Diego, CA 92121 USA; 8 Hexal AG, Industriestraße 25, D-83607 Holzkirchen, Germany; 9 Pfizer UK, Discovery Park, Ramsgate Road, Sandwich, CT13 9ND UK; 10 Pfizer Inc., 300 Technology Square, Cambridge, MA 02139 USA

**Keywords:** Infliximab, PF-06438179/GP1111, Biosimilar, Rheumatoid arthritis, Dose escalation

## Abstract

**Background:**

This double-blind, active-controlled, randomized, multinational study evaluated the efficacy, safety, pharmacokinetics (PK), and immunogenicity of PF-06438179/GP1111 (IxifiTM/Zessly®), an infliximab biosimilar, vs infliximab (Remicade®) reference product sourced from the European Union (infliximab-EU) in biologic-naïve patients with moderate to severe active rheumatoid arthritis (RA) despite methotrexate therapy. This paper reports results from the initial 30-week treatment period.

**Methods:**

Patients (*N* = 650) were stratified by geographic region and randomized 1:1 to PF-06438179/GP1111 or infliximab-EU (3 mg/kg intravenous at weeks 0, 2, and 6, then every 8 weeks). Dose escalation to 5 mg/kg was allowed starting at week 14 for patients with inadequate RA response. The primary endpoint was American College of Rheumatology criteria for ≥ 20% clinical improvement (ACR20) response at week 14. Therapeutic equivalence was declared if the two-sided 95% CI for the treatment difference was within the symmetric equivalence margin of ± 13.5%. Statistical analysis was also performed with a two-sided 90% CI using an asymmetric equivalence margin (− 12.0%, 15.0%).

**Results:**

Patients (80.3% female; 79.4% seropositive) had a mean RA duration of 6.9 years, and mean baseline Disease Activity Score in 28 joints, four components based on C-reactive protein was 6.0 in both arms. Week 14 ACR20 in the intention-to-treat population was 62.7% for PF-06438179/GP1111 and 64.1% for infliximab-EU. Week 14 ACR20 using nonresponder imputation was 61.1% for PF-06438179/GP1111 and 63.5% for infliximab-EU, and the 95% (− 9.92%, 5.11%) and 90% (− 8.75%, 4.02%) CIs for the treatment difference (− 2.39%) were entirely contained within the prespecified symmetric and asymmetric equivalence margins, respectively. No differences were observed between arms for secondary efficacy endpoints. Overall postdose antidrug antibody (ADA) rates through week 30 were 48.6% and 51.2% for PF-06438179/GP1111 and infliximab-EU, respectively. Efficacy and immunogenicity were similar between treatments for patients with dose escalation (at or after week 14), as well as between treatments for patients without dose escalation. Safety profiles of PF-06438179/GP1111 and infliximab-EU were similar, with no clinically meaningful differences observed between arms, including after ADA development. Serum drug concentrations were similar between arms at each time point during the initial 30-week treatment period.

**Conclusion:**

PF-06438179/GP1111 and infliximab-EU demonstrated similar efficacy, safety, immunogenicity, and PK with or without dose escalation in patients with moderate to severe active RA on background methotrexate.

**Trial registration:**

ClinicalTrials.gov, NCT02222493. Registered on 21 August 2014.

EudraCT, 2013-004148-49. Registered on 14 July 2014.

**Electronic supplementary material:**

The online version of this article (10.1186/s13075-018-1646-4) contains supplementary material, which is available to authorized users.

## Background

Infliximab (Remicade®; Janssen Biotech, Horsham, PA, USA, and Janssen Biologics B.V., Leiden, The Netherlands) is a chimeric monoclonal antibody specific for human tumor necrosis factor (TNF)-α, a cytokine with a demonstrated role in autoimmune and inflammatory diseases [1, 2]. Infliximab, in combination with methotrexate (MTX), is indicated for the reduction of signs and symptoms of moderate to severe active rheumatoid arthritis (RA) [1, 2]. Among patients with RA, those who receive infliximab plus MTX achieve greater clinical, radiographic, and functional benefits than those who receive MTX alone [3, 4]. Nevertheless, access to infliximab varies. Differences in national reimbursement criteria between European countries have created inequities in access to biologic disease-modifying antirheumatic drugs (DMARDs), such as infliximab, for patients with RA [5]. Furthermore, patients in the United States with RA who are covered by Medicaid are less likely than privately insured patients to receive biologic DMARDs, demonstrating disparities in treatment access by insurance type [6].

A biosimilar is a biologic drug that is highly similar in structure and function to a licensed (i.e., reference or originator) biologic product [7, 8]. In defining a biosimilar, the U.S. Food and Drug Administration further specifies that there must be no clinically meaningful differences in safety, purity, and potency between the biosimilar and the reference products [8]. The European Medicines Agency requires evidence to demonstrate the similar nature of the two products in terms of quality, safety, and efficacy [7]. The introduction of biosimilars has been associated with cost savings and improved access to biologic therapies [9–11]. For example, introduction of epoetin biosimilars in the European Union (EU) was followed by a 27% decrease in treatment costs and a 16% increase in the use of erythropoietins [9]. Furthermore, a budget impact analysis of switching patients to the infliximab biosimilar CT-P13 projected annual cost savings that could support treatment for an additional 1960 (10% price discount) to 7561 (30% price discount) patients across France, Germany, Italy, the Netherlands, and the United Kingdom [10]. This is complemented by real-world data from the NOR-SWITCH trial, which demonstrated that switching from originator infliximab (Remicade®) to CT-P13 on the basis of cost was not inferior to continued treatment with originator infliximab in patients with chronic inflammatory diseases [12].

PF-06438179/GP1111 (IxifiTM/Zessly®; Pfizer Inc, New York, NY, USA, and Sandoz GmbH, Kundl, Austria) is a biosimilar of infliximab (Remicade®) reference product marketed in the United States (infliximab-US) and the EU (infliximab-EU) [13, 14]. Comparative assessments of protein structure confirmed that PF-06438179/GP1111 and infliximab have an identical primary amino acid sequence and similar posttranslational modifications, charge heterogeneity, and product purity [15]. In vitro characterization of biological activity established functional similarity in the ability of PF-06438179/GP1111 and infliximab to bind TNF and inhibit TNF-induced cell apoptosis [15]. PF-06438179/GP1111 demonstrated similar toxicokinetic, tolerability, and antidrug antibody (ADA) responses to infliximab in a nonclinical in vivo toxicity study [15]. A phase I pharmacokinetics (PK) clinical study in healthy volunteers demonstrated similarity in PK, safety, and immunogenicity of PF-06438179/GP1111 to infliximab-US and infliximab-EU, as well as between infliximab-EU and infliximab-US reference products [16]. In the present double-blind, active-controlled, randomized, multinational study, we compared the efficacy, safety, PK, and immunogenicity of PF-06438179/GP1111 with infliximab-EU (Remicade®), each with background MTX therapy, as treatment for patients with moderate to severe active RA and inadequate response to MTX therapy. We report the efficacy and safety results from the initial 30-week treatment period.

## Methods

### Study population

Eligible patients were adults (aged ≥18 years) who met the 2010 American College of Rheumatology/European League Against Rheumatism (ACR/EULAR) classification criteria for RA for ≥ 4 months and ACR classes I–III functional status, based on the 1991 revised criteria [17, 18]. Patients had moderate to severe active RA, with at least six swollen and at least six tender joints at both screening and baseline, and high-sensitivity C-reactive protein (hs-CRP) ≥ 10 mg/L at screening. Patients must have received oral or parenteral MTX (10–25 mg/wk) for ≥ 12 weeks (at stable dose for ≥ 4 weeks) and oral folic/folinic acid (≥ 5 mg/wk) for ≥ 21 days prior to the first dose of study drug. Patients intolerant to 10–25 mg/wk could enroll with an MTX dose as low as 7.5 mg/wk. A dose of 6.0 mg/wk was allowed in geographic regions where specified by local guidance or standard of care.

Patients enrolled under the original protocol could receive concomitant sulfasalazine and/or antimalarial drugs at a stable dose. A protocol amendment later removed these allowable background therapies and required a 4-week washout period prior to the first dose of study drug. Use of other DMARDs also required a washout period prior to the first dose of study drug.

The main exclusion criteria were current infection or infection requiring hospitalization or parenteral antimicrobial therapy judged clinically significant by the investigator ≤ 6 months prior to the first dose of study drug; evidence or history of congestive heart failure, demyelinating disease, untreated or inadequately treated latent or active tuberculosis, or malignancy within the past 5 years; inadequate bone marrow, liver, renal, and immune system function at screening; and positivity for human immunodeficiency virus, hepatitis B virus, or hepatitis C virus. Patients were excluded if they had current or prior treatment with infliximab or lymphocyte-depleting therapies (e.g., rituximab, alemtuzumab); however, they were allowed up to two doses of one nondepleting, noninfliximab biologic if discontinued ≥ 12 weeks or five half-lives (whichever was longer) prior to the first dose of study drug.

### Study design and treatments

**Fig. 1:**
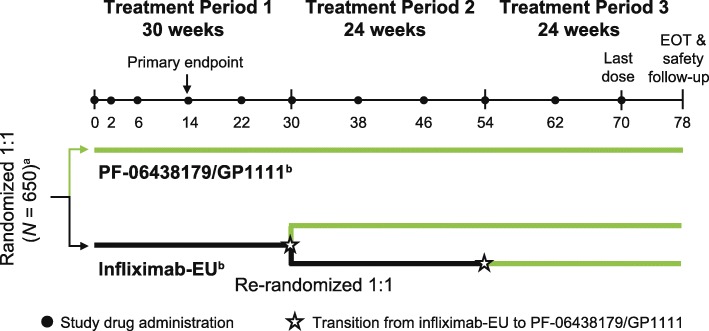
Study design. ^a^A sample size of approximately 614 patients was planned for enrollment; the actual number of patients randomized was 650. ^b^Intravenous PF-06438179/GP1111 or infliximab-EU 3 mg/kg was given as an induction regimen at weeks 0, 2, and 6, followed by maintenance treatment with a 3 mg/kg dose starting at week 14 and continuing every 8 weeks thereafter. Dose escalation to 5 mg/kg PF-06438179/GP1111 or infliximab-EU was permitted at or after week 14 for patients with inadequate RA response. *EOT* End of treatment, *Infliximab-EU* Infliximab sourced from the European Union, *RA* Rheumatoid arthritis

This double-blind, active-controlled, randomized, multinational study (ClinicalTrials.gov identifier NCT02222493; EudraCT number 2013-004148-49) [19] was initiated at 174 centers in 28 countries. The study consisted of an initial 30-week treatment period (treatment period 1) and two subsequent 24-week treatment periods, during which patients were evaluated following a single transition from infliximab-EU to PF-06438179/GP1111 after 30 (treatment period 2) or 54 (treatment period 3) weeks of treatment (Fig. 1). At the start of treatment period 1, patients were randomized (1:1) to receive blinded treatment with PF-06438179/GP1111 or infliximab-EU, each in combination with MTX, with randomization stratified according to geographic region (North America and Western Europe, Japan, Republic of Korea, Latin America, and the rest of the world). At the start of treatment period 2, patients on infliximab-EU were rerandomized (1:1) to either blinded treatment with continued infliximab-EU or a transition to PF-06438179/GP1111. During treatment period 3, all patients received open-label treatment with PF-06438179/GP1111. This report presents the efficacy and safety results for treatment period 1, which ended with the week 30 predose assessment.

PF-06438179/GP1111 or infliximab-EU solutions for infusion were prepared by the site’s pharmacists, who were designated to participate in the study and unblinded with regard to study treatments. Intravenous infliximab (PF-06438179/GP1111 or infliximab-EU) 3 mg/kg was given as an induction regimen at weeks 0, 2, and 6, followed by maintenance treatment with a 3 mg/kg dose starting at week 14 and continuing every 8 weeks thereafter. Dose escalation to 5 mg/kg infliximab was allowed starting at week 14 for patients who failed to achieve ≥ 20% improvement from baseline in both tender (68) and swollen (66) joint counts. Dose escalation to 5 mg/kg infliximab was also allowed for patients who achieved this response at week 14 but subsequently lost response to < 20% improvement from baseline in both joint counts. Patients remained on the escalated dose level for the remainder of the study. Premedication with antihistamines, acetaminophen/paracetamol, and/or corticosteroids could be administered at the investigator’s discretion in compliance with local practice, the premedication label, and regulations.

Patients were required to continue their stable background MTX dose (10–25 mg/wk, 7.5 mg/wk if intolerant to higher doses, or 6 mg/wk in geographic regions where specified by local guidance or standard of care), any second DMARD (sulfasalazine/hydroxychloroquine), and folic/folinic acid supplementation throughout the study. If receiving corticosteroid (≤ 10 mg/d prednisone equivalent) and/or a nonsteroidal anti-inflammatory drug/Cox-2 inhibitor, the stable background dose remained the same for the first year, unless toxicity occurred.

### Primary and secondary efficacy endpoints

The primary efficacy endpoint was the percentage of patients achieving ACR criteria for ≥ 20% clinical improvement (ACR20) at week 14. Evaluation of ACR20 response at this time point reflects the beginning of the therapeutic plateau and provides greater sensitivity to detect possible differences in the rate of response between treatment arms, as compared with later time points [20].

Secondary efficacy endpoints at weeks 2, 4, 6, 12, 14, 22, and 30 included ACR20 (other than week 14), ACR50 (≥ 50% clinical improvement), and ACR70 (≥ 70% clinical improvement) response rates; Disease Activity Score in 28 joints, four components based on C-reactive protein (DAS28-CRP); percentages of patients with response defined according to EULAR criteria; the percentages of patients with DAS and ACR/EULAR remission; and changes from baseline for individual ACR parameters, including Health Assessment Questionnaire Disability Index (HAQ-DI). Patients were considered to be in DAS remission when DAS28-CRP was < 2.6, and in ACR/EULAR remission when either scores for tender joint count, swollen joint count, hs-CRP, and patient global assessment were all ≤ 1, or when the Simplified Disease Activity Index score was ≤ 3.3. Joint examinations were performed by an independent assessor who was blinded with regard to study treatments. In addition, pharmacodynamic (PD) response was assessed by the serum hs-CRP concentration (Covance Inc., Princeton, NJ, USA).

### Additional secondary endpoints

Safety endpoints included adverse events (AEs) and laboratory abnormalities, characterized by their type, incidence, severity, timing, duration, seriousness, and relatedness to study drug. Other safety measures included electrocardiogram readings, vital signs, and physical examination. AEs were coded using the Medical Dictionary for Regulatory Activities (MedDRA; version 19.0) classification system, and severity was graded according to the National Cancer Institute Common Terminology Criteria for Adverse Events (version 4.03). Treatment-emergent AEs (TEAEs) were defined as any AE that occurred, or any preexisting AE that worsened, after the beginning of study treatment. TEAEs of special interest comprised infusion-related reactions (IRRs), hypersensitivity, infections (including tuberculosis and pneumonia), and malignancy (including lymphoma). Hypersensitivity events were identified by applying the MedDRA version 19.0 search criteria, hypersensitivity standardized MedDRA query (broad and narrow), and anaphylactic reactions standardized MedDRA query (broad and narrow), and high-level group terms immunology and allergy investigations.

Immunogenicity endpoints included the incidence and titers of ADAs and neutralizing antibodies (NAbs). Serum samples were first analyzed at ICON Laboratory Services, Inc. (Whitesboro, NY, USA) for the presence of ADAs using a validated electrochemiluminescence assay with a tiered approach of screening, confirmation, and titer/quantitation. ADA-positive samples were those that tested positive at both screening and confirmation, and had an ADA titer ≥ 1.30. Confirmed ADA-positive samples were then tested for NAbs using a validated cell-based bioassay with a tiered approach of screening and titer/quantitation. NAb-positive samples were those that tested positive at screening and had an NAb titer ≥ 0.70. The criteria for defining positive and negative results was established as cut points during method validation against the biosimilar for ADA and NAb assays. Transient ADA response was defined as having treatment-induced ADA detected at at least two sampling time points during treatment (including the follow-up period), where the first and last ADA-positive samples (regardless of any negative samples in between) were separated by < 16 weeks and the patient’s last sampling time point was ADA-negative.

PK serum samples were analyzed for PF-06438179/GP1111 and infliximab-EU at ICON Laboratory Services, Inc. using a validated, sensitive, and specific enzyme-linked immunosorbent assay with limits of quantification of 100 ng/ml (lower) and 5000 ng/ml (upper).

### Statistical methods

A sample of 614 patients was planned; this provided ≥ 85% power to demonstrate equivalence using a prespecified symmetric margin of ± 13.5% with a two-sided 95% CI when assuming ACR20 response rates of 57.5% at week 14 in both arms. The symmetric equivalence margin (± 13.5%) with a two-sided 95% CI was derived using a meta-analysis of historical published data for infliximab in RA, and > 50% preservation of the historical treatment effect for infliximab as compared with placebo [21–26]. In addition, an asymmetric margin of − 12.0% to 15.0% with a two-sided 90% CI was specifically requested by the U.S. Food and Drug Administration.

Primary analysis of ACR20 response rate at week 14 was performed in the intention-to-treat (ITT) population (all randomized patients) using nonresponder imputation (NRI) for missing data and for patients who discontinued study treatment prior to week 14. Robustness of the primary analysis was confirmed for the per-protocol (PP) population (all patients who received study treatment as planned up to week 14 and had no major protocol deviations). Other sensitivity analyses for the primary endpoint included analysis using observed data, analysis adjusting for the stratification variable geographic region, analysis incorporating one additional responder in the infliximab-EU arm who was identified at site closeout, and tipping point analysis for the asymmetric margin (− 12.0%, 15.0%) based on multiple imputation of missing data (Additional file 1: Supplementary Methods). In addition, repeated measures analysis of ACR20 response rates across all study visits up to week 30 was performed, adjusting for geographic region. The analysis was performed using the original database snapshot for the week 30 clinical study report and an additional sensitivity analysis for the one additional responder identified at site closeout.

Secondary efficacy endpoints were summarized descriptively; no conclusions regarding equivalence were drawn from analyses of secondary endpoints. Safety and immunogenicity endpoints were analyzed descriptively for the safety population (all randomized patients who received at least a portion of at least one dose of study drug). Drug concentration–time data were summarized descriptively for the PK population (all patients from the safety population who provided at least one postdose drug concentration measurement).

## Results

### Patient disposition and demographics

A total of 1603 patients were screened, of whom 650 were randomized to study treatment. The most common reason for screening failure was low hs-CRP value. The ITT population included 324 and 326 patients in the PF-06438179/GP1111 and infliximab-EU study arms, respectively (Additional file 1: Figure. S1). One patient in the PF-06438179/GP1111 arm was randomized twice; data were not collected for this patient’s second randomization. Therefore, the safety population consisted of 323 (99.7%) patients who received PF-06438179/GP1111 and 326 (100%) who received infliximab-EU. A total of 280 (86.4%) patients in the PF-06438179/GP1111 arm and 286 (87.7%) in the infliximab-EU arm completed the 30-week treatment period. Forty-three (13.3%) patients in the PF-06438179/GP1111 arm and 40 (12.3%) in the infliximab-EU arm discontinued treatment, including 23 (7.1%) and 13 (4.0%), respectively, who discontinued before week 14.

Patient demographics and baseline disease characteristics were similar between treatment arms (Tables 1 and 2). Within each geographic region, enrollment in the two study arms was completely balanced or varied by only one patient because randomization was stratified by region. The majority of all patients in the ITT population were women (80.3%) and rheumatoid factor- or anticyclic citrullinated peptide antibody-positive (79.4%). Patients were biologic-naïve, defined as receipt of up to two doses of one prior noninfliximab, nondepleting biologic DMARD; only ten (1.5%) had received a biologic DMARD, of whom four exceeded the two doses maximally allowed of one prior biologic. Eleven (1.7%) patients received concomitant sulfasalazine or antimalarial drugs under the original protocol. Patients had a mean RA duration of 6.9 years, with a mean of 16.2 swollen and 25.2 tender joints, a mean DAS28-CRP of 6.0, and a mean hs-CRP of 25.6 mg/L. The mean dose of MTX and the percentage of patients receiving oral corticosteroids were similar between the two arms (Table 2).

**Table 1:** Patient demographic characteristics (intention-to-treat population)

	PF-06438179/GP1111 (*n* = 324)	Infliximab-EU (*n* = 326)	All patients (*N* = 650)
Gender, *n* (%)			
Female	258 (79.4)	264 (81.0)	522 (80.3)
Male	66 (20.4)	62 (19.0)	128 (19.7)
Age, mean (SD), years	52.8 (13.3)	52.8 (12.9)	52.8 (13.1)
Weight, mean (SD), kg	73.3 (19.8)	74.2 (20.0)	73.8 (19.9)
Body mass index, mean (SD), kg/m^2^	27.2 (6.4)	27.7 (7.0)	27.4 (6.7)
Race, *n* (%)			
White	257 (79.3)	247 (75.8)	504 (77.5)
Black	5 (1.5)	9 (2.8)	14 (2.2)
Asian	46 (14.2)	45 (13.8)	91 (14.0)
Other	15 (4.6)	25 (7.7)	40 (6.2)
Unspecified	1 (0.3)	0	1 (0.2)
Geographic region, *n* (%)			
North American and Western Europe	50 (15.4)	51 (15.6)	101 (15.5)
Japan	24 (7.4)	23 (7.1)	47 (7.2)
South Korea	4 (1.2)	5 (1.5)	9 (1.4)
Latin America	22 (6.8)	22 (6.7)	44 (6.8)
Rest of the world	224 (69.1)	225 (69.0)	449 (69.1)

*Infliximab-EU* Infliximab sourced from the European Union

**Table 2:** Baseline disease characteristics (intention-to-treat population)

	PF-06438179/GP1111 (*n* = 324)	Infliximab-EU (*n* = 326)	All patients (*N* = 650)
RA duration, mean (SD), years	7.3 (8.6)	6.4 (6.7)	6.9 (7.7)
RF or anti-CCP antibody positive, *n* (%)	249 (76.9)	267 (81.9)	516 (79.4)
Swollen joint count, mean (SD)	16.1 (9.4)	16.3 (8.7)	16.2 (9.1)
Tender joint count, mean (SD)	24.7 (13.9)	25.7 (12.9)	25.2 (13.4)
hs-CRP, mg/L			
Mean (SD)	25.8 (24.3)	25.3 (28.4)	25.6 (26.4)
Median (range)	17.9 (0.5–135.0)	16.5 (0.8–203.0)	17.4 (0.5–203.0)
DAS28-CRP, mean (SD)	6.0 (1.0)	6.0 (0.9)	6.0 (0.9)
HAQ-DI, mean (SD)	1.6 (0.6)	1.6 (0.7)	NC
Prior use of one biologic drug, *n* (%)	7 (2.2)^a^	3 (0.9)	10 (1.5)^a^
MTX dose, mean (SD), mg/wk	14.2 (4.5)^b^	14.4 (4.5)	14.3 (4.5)^b^
Corticosteroid use, *n* (%)	178 (54.9)	192 (58.9)	370 (56.9)
Antimalarial drug use,^c^ *n* (%)	2 (0.6)	5 (1.5)	7 (1.1)
Sulfasalazine drug use,^c^ *n* (%)	2 (0.6)	2 (0.6)	4 (0.6)

*Abbreviations: Anti-CCP* Anticyclic citrullinated peptide, *DAS28-CRP* Disease Activity Score in 28 joints, four components based on C-reactive protein, *HAQ-DI* Health Assessment Questionnaire Disability Index, *hs-CRP* High-sensitivity C-reactive protein, *Infliximab-EU* Infliximab sourced from the European Union, *MTX* Methotrexate, *NC* not calculated, *RA* Rheumatoid arthritis, *RF* Rheumatoid factor

^a^Includes one patient (PF-06438179/GP1111) who received more than two doses of sarilumab; this patient was not captured as biologic-experienced but was correctly recorded as having an exclusion criterion protocol deviation

^b^Total weekly dose of MTX was 16 mg/wk for one patient (PF-06438179/GP1111) but incorrectly recorded as 32 mg/wk; incorrect dose was the maximum value of the MTX dose range and was used for calculation of mean dose

^c^Use of sulfasalazine and antimalarial drugs was allowed only in the original protocol, but not in subsequent protocol amendments

Total dose exposure and the percentage of patients with dose escalation to 5 mg/kg at or after week 14 were similar between the two arms. Sixty (18.5%) and 68 (20.9%) patients in the PF-06438179/GP1111 and infliximab-EU arms, respectively, had dose escalation to 5 mg/kg at week 14. An additional 23 (7.1%) and 15 (4.6%) patients in the PF-06438179/GP1111 and infliximab-EU arms, respectively, had dose escalation to 5 mg/kg at week 22.

### Efficacy

**Fig. 2:**
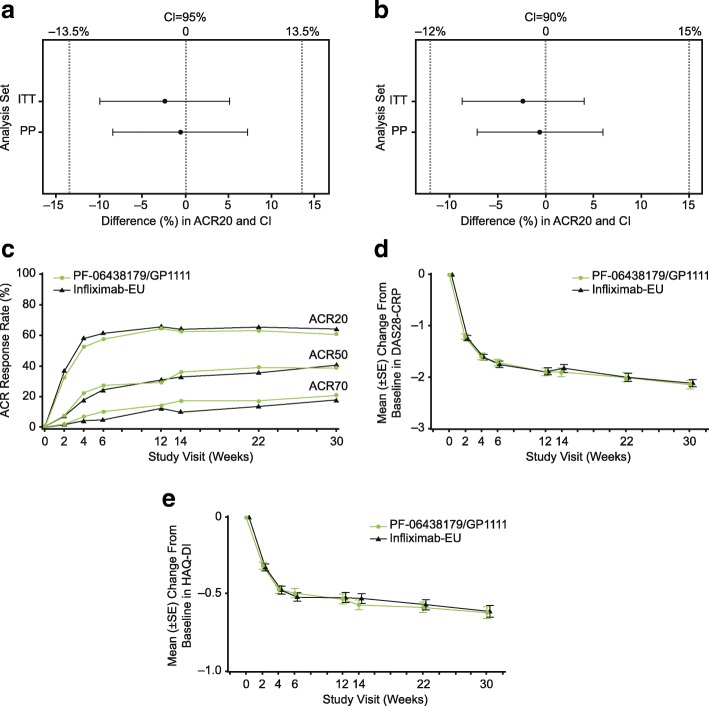
Efficacy of PF-06438179/GP1111 and infliximab-EU. **a** Difference (95% CI) in week 14 ACR20 response between PF-06438179/GP1111 and infliximab-EU using NRI and symmetric equivalence margin. **b** Difference (90% CI) in week 14 ACR20 response between PF-06438179/GP1111 and infliximab-EU using NRI and asymmetric margin. **c** ACR20, ACR50, and ACR70 response rates by visit (ITT population). **d** Mean (± SE) change from baseline in DAS28-CRP by visit (ITT population). **e** Mean (± SE) change from baseline in HAQ-DI by visit (ITT population). *ACR20/50/70* American College of Rheumatology criteria for ≥ 20%/50%/70% clinical improvement, *DAS28-CRP* Disease Activity Score in 28 joints, four components based on C-reactive protein, *HAQ-DI* Health Assessment Questionnaire Disability Index; *Infliximab-EU* Infliximab sourced from the European Union, *ITT* Intention to treat, *NRI* Nonresponder imputation, *PP* Per protocol

In the ITT population, 203 (62.7%) patients in the PF-06438179/GP1111 arm and 209 (64.1%) in the infliximab-EU arm achieved ACR20 response at week 14. Using NRI (required for 18 [5.6%] and 12 [3.7%] PF-06438179/GP1111 and infliximab-EU patients, respectively), 198 (61.1%) patients in the PF-06438179/GP1111 arm and 207 (63.5%) in the infliximab-EU arm had a week 14 ACR20 response. The treatment difference was − 2.39%, and the corresponding 95% (− 9.92%, 5.11%) and 90% (− 8.75%, 4.02%) CIs were entirely contained within the prespecified symmetric (± 13.5%) and asymmetric (− 12.0%, 15.0%) equivalence margins, respectively (Fig. 2a and b).

ACR20 response rates at week 14 for the PP population were similar to those reported for the ITT population for both PF-06438179/GP1111 (186 of 279 [66.7%]) and infliximab-EU (195 of 290 [67.2%]). Furthermore, the 95% (− 8.42%, 7.23%) and 90% (− 7.15%, 6.02%) CIs for the treatment difference of − 0.58% were entirely contained within the prespecified symmetric and asymmetric equivalence margins, respectively (Fig. 2a and b). Other sensitivity analyses of the primary endpoint were consistent with the primary analysis results (Additional file 1: Supplementary Results, Table S1, Figure S2).

ACR20, ACR50, and ACR70 response rates were similar between PF-06438179/GP1111 and infliximab-EU at all time points through week 30 (Fig. 2c; Additional file 1: Supplementary Results; Table S2). Mean change from baseline in DAS28-CRP (Fig. 2d) was similar between PF-06438179/GP1111 and infliximab-EU at each study visit. At week 30, a mean decrease of 2.1 in DAS28-CRP from baseline was observed for both arms. Likewise, the percentages of patients in each EULAR response category were similar between treatment arms (Additional file 1: Table S3). At week 30, 31.2% of PF-06438179/GP1111 and 28.8% of infliximab-EU patients a achieved good EULAR response.

Similar percentages of patients in the PF-06438179/GP1111 and infliximab-EU arms achieved DAS or ACR/EULAR remission at each study visit (Additional file 1: Table S4). At week 30, DAS remission was achieved by 19.1% and 16.6% of PF-06438179/GP1111 and infliximab-EU patients, respectively. ACR/EULAR remission was achieved by 9.3% and 7.1% of patients, respectively, including 6.8% and 5.5% using the Boolean definition. Mean values and changes from baseline in HAQ-DI (Fig. 2e) were similar between the two treatment arms at each study visit up to week 30. The maximal decrease in HAQ-DI was observed at week 30, with a mean decrease of 0.6 from baseline observed for both arms. Likewise, mean values and changes from baseline in the PD marker hs-CRP were similar between the PF-06438179/GP1111 and infliximab-EU arms at each study visit (Additional file 1: Figure S3), with a maximum decrease from baseline at week 2 for both PF-06438179/GP1111 (17.2 mg/L) and infliximab-EU (16.1 mg/L).

ACR20 responses were similar between PF-06438179/GP1111 and infliximab-EU for patients who dose-escalated to 5 mg/wk at week 14 and between arms for patients who did not (Table 3). ACR20 response rates at week 30 for PF-06438179/GP1111 and infliximab-EU patients who dose-escalated at week 14 were 45.0% and 39.7%, respectively, and were 6 of 23 (26.1%) and 7 of 15 (46.7%) respectively, for patients who dose-escalated at week 22.

**Table 3:** Descriptive summary of ACR20 response rate at weeks 22 and 30 by dose received at week 14 (intention-to-treat population)

	No dose escalation (3 mg/kg) at week 14			Dose escalation (5 mg/kg) at week 14		
	PF-06438179/GP1111 (*n* = 240), *n* (%)	Infliximab-EU (*n* = 244), *n* (%)	Treatment difference, %	PF-06438179/GP1111 (*n* = 60), *n* (%)	Infliximab-EU (*n* = 68), *n* (%)	Treatment difference, %
Week 22						
ACR20 response						
Yes	180 (75.0)	185 (75.8)	− 0.82	23 (38.3)	27 (39.7)	− 1.37
No	58 (24.2)	58 (23.8)		36 (60.0)	36 (52.9)	
Missing	2 (0.8)	1 (0.4)		1 (1.7)	5 (7.4)	
Week 30						
ACR20 response						
Yes	169 (70.4)	181 (74.2)	− 3.76	27 (45.0)	27 (39.7)	5.29
No	65 (27.1)	55 (22.5)		29 (48.3)	30 (44.1)	
Missing	6 (2.5)	8 (3.3)		4 (6.7)	11 (16.2)	

*ACR20* American College of Rheumatology criteria for ≥ 20% clinical improvement, *Infliximab-EU* Infliximab sourced from the European Union

ACR20 response rates at weeks 14 and 30 trended higher for the patient subset that did not develop an ADA through week 30 (PF-06438179/GP1111, *n* = 220; infliximab-EU, *n* = 222) as compared with the ADA-positive subset (PF-06438179/GP1111, *n* = 100; infliximab-EU, *n* = 103), but they were similar between the two treatment arms within each subset. At week 14, 152 (69.1%) and 158 (71.2%) patients in the PF-06438179/GP1111 and infliximab-EU ADA-negative subsets, respectively, had ACR20 response, as compared with 51 (51.0%) and 51 (49.5%) patients, respectively, in the ADA-positive subsets.

### Safety

A total of 185 (57.3%) patients in the PF-06438179/GP1111 arm and 176 (54.0%) in the infliximab-EU arm reported all-cause TEAEs (Table 4). The MedDRA System Organ Class (SOC) with the highest percentage of patients was infections and infestations in 86 (26.6%) and 72 (22.1%) PF-06438179/GP1111 and infliximab-EU patients, respectively. The most frequently reported TEAE was IRR in 19 (5.9%) and 21 (6.4%) patients in the PF-06438179/GP1111 and infliximab-EU arms, respectively. In the PF-06438179/GP1111 and infliximab-EU arms, respectively, 31 (9.6%) and 28 (8.6%) patients temporarily discontinued, and 23 (7.1%) and 24 (7.4%) patients permanently discontinued, treatment due to AEs. Sixteen (5.0%) and 14 (4.3%) patients, respectively, discontinued study participation because of AEs, including 4 (1.2%) and 3 (0.9%) due to IRR.

**Table 4:** All-cause treatment-emergent adverse events (safety population)^a^

	PF-06438179/GP1111 (*n* = 323)	Infliximab-EU (*n* = 326)
Number of AEs	486	492
Patients with events, *n* (%)		
AEs	185 (57.3)	176 (54.0)
SAEs	16 (5.0)	20 (6.1)
Grade 3 AEs	34 (10.5)	34 (10.4)
Grade 4 AEs	1 (0.3)	6 (1.8)
Grade 5 AEs	2 (0.6)	1 (0.3)
Temporarily discontinued from treatment due to AEs	31 (9.6)	28 (8.6)
Permanently discontinued from treatment due to AEs	23 (7.1)	24 (7.4)
Discontinued from study due to AEs	16 (5.0)	14 (4.3)

*Abbreviations: AE* Adverse event, *Infliximab-EU* Infliximab sourced from the European Union, *SAE* Serious adverse event

^a^Includes all AEs collected from the first infusion through week 30 study visit for each patient. AEs were graded in accordance with National Cancer Institute Common Terminology Criteria for AEs (version 4.03). Grades 1–5 AEs are defined as mild, moderate, severe, and life-threatening AEs and death related to AEs, respectively

TEAEs reported by the investigator as potentially related to study treatment (treatment-related) occurred in 81 (25.1%) and 75 (23.0%) patients in the PF-06438179/GP1111 and infliximab-EU arms, respectively. The SOC with the highest percentage of patients who experienced treatment-related TEAEs was infections and infestations in 28 (8.7%) and 22 (6.7%) PF-06438179/GP1111 and infliximab-EU patients, respectively. The most frequently reported treatment-related TEAE was IRR in 17 (5.3%) and 20 (6.1%) PF-06438179/GP1111 and infliximab-EU patients, respectively.

Sixteen (5.0%) patients in the PF-06438179/GP1111 arm and 20 (6.1%) in the infliximab-EU arm reported serious adverse events (SAEs) (Table 4). The two SOCs with the highest percentages of patients who experienced all-cause SAEs (PF-06438179/GP1111 vs infliximab-EU) were infections and infestations (six [1.9%] vs nine [2.8%]) and cardiac disorders (four [1.2%] vs three [0.9%]). Two patients in each treatment arm experienced SAEs with a fatal outcome; one death (infliximab-EU) following an SAE that started in treatment period 1 occurred outside the 30-week treatment period.

TEAEs of special interest included the IRRs noted above and hypersensitivity events in 44 (13.6%) and 51 (15.6%) patients in the PF-06438179/GP1111 and infliximab-EU arms, respectively (Additional file 1: Table S5). IRRs and hypersensitivity events occurring on or after the date a patient first tested positive for ADAs were similar between treatment arms, with 11 (7.0%) PF-06438179/GP1111 and 14 (8.4%) infliximab-EU patients experiencing a single IRR and 11 (7.0%) PF-06438179/GP1111 and 19 (11.4%) infliximab-EU patients reporting 14 and 25 hypersensitivity events, respectively. In summary, the majority (25 of 40 patients) of IRR events in both arms appeared to be associated with the development of ADAs, whereas the correlation for the broader category of hypersensitivity events, which included IRRs (30 of 95 patients), appeared to be weaker.

Treatment-emergent infectious AEs were reported by 87 (26.9%) and 73 (22.4%) patients in the PF-06438179/GP1111 and infliximab-EU arms, respectively (Additional file 1: Table S5). Six patients (three [0.9%] per arm) reported pneumonia as follows: two cases of pneumonia and one case of *Pneumocystis jirovecii* pneumonia in the PF-06438179/GP1111 arm, and three cases of pneumonia in the infliximab-EU arm. One (0.3%) patient in the PF-06438179/GP1111 arm reported latent tuberculosis, and one (0.3%) patient in the infliximab-EU arm reported active tuberculosis. One (0.3%) patient in each arm reported malignant tumors (colon cancer). The percentages of patients with laboratory abnormalities and the severity of abnormalities as well as vital sign results were comparable between treatment arms.

### Immunogenicity and PK

**Fig. 3:**
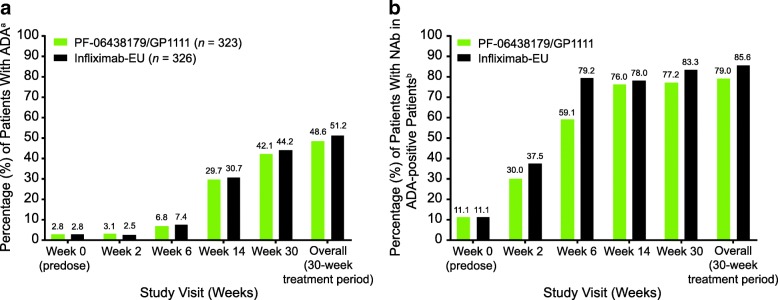
ADA and NAb incidence by study visit (safety population). **a** ADA incidence. **b** NAb incidence. ^a^ADA-positive and ADA-negative test results were defined as ADA titer ≥ 1.30 and < 1.30, respectively. Overall, a patient who tested positive was defined as having at least one postdose positive sample during the 30-week treatment period, regardless of predose ADA status. ^b^NAb-positive and NAb-negative results were defined as NAb titer ≥ 0.70 and < 0.70, respectively. Incidences of NAb-positive patients are expressed as percentages of ADA-positive patients. *ADA* Antidrug antibody; *Infliximab-EU* Infliximab sourced from the European Union, *NAb*, Neutralizing antibody

The incidence of ADA was similar between treatment arms at all measured time points (Fig. 3a; Additional file 1: Table S6). At baseline, nine (2.8%) patients in each of the PF-06438179/GP1111 and infliximab-EU arms tested positive for ADA, with five (55%) and two (22%) of these patients, respectively, also testing positive at week 2. Overall, 157 (48.6%) and 167 (51.2%) patients in the PF-06438179/GP1111 and infliximab-EU arms, respectively, had at least one postdose sample that tested positive for ADA during the 30-week treatment period. Only one patient (0.6%) in each arm had a transient ADA response. The distribution of ADA titers was comparable between arms over the 30-week treatment period (Additional file 1: Figure S4). Of the ADA-positive patients, 124 (79.0%) and 143 (85.6%), in the PF-06438179/GP1111 and infliximab-EU arms, respectively, tested NAb-positive. The incidence of NAb was similar between treatment arms at all measured time points. (Fig. 3b; Additional file 1: Table S6)

Patients qualifying for dose escalation to 5 mg/kg infliximab had higher ADA rates of 38.6% (PF-06438179/GP1111) and 44.6% (infliximab-EU) at week 14, as compared with 26.7% (PF-06438179/GP1111) and 25.9% (infliximab-EU) for patients remaining on 3 mg/kg (Additional file 1: Table S7). Incidence of ADA was similar between PF-06438179/GP1111 and infliximab-EU for patients who dose-escalated, as well as between the two arms for patients who did not (Additional file 1: Table S7). A similar trend was observed for NAb rates between treatment arms for patients who remained at 3 mg/kg infliximab. There were slight numerical differences in NAb rates between treatment arms for patients who dose-escalated to 5 mg/kg infliximab, which could be due to smaller subgroup size. However, these differences are not clinically meaningful, because ACR20 response rates were similar between treatment arms for patients who dose-escalated to 5 mg/kg infliximab.

Trough serum concentrations at weeks 2, 4, 6, 14, 22, and 30 and immediate postdose serum concentrations on day 1 and week 14 were similar between treatment arms (Table 5). The PK of infliximab is known to be affected by the presence of ADA [2], and the serum drug concentrations in this trial were lower in ADA-positive than in ADA-negative patients (Table 5). The presence of ADA affected the disposition of both PF-06438179/GP1111 and infliximab-EU in a similar manner in ADA-positive patients (Table 5; Additional file 1: Figure S5).

**Table 5:** Serum PF-06438179/GP1111/GP1111 and infliximab-EU concentrations by study visit and antidrug antibody status (pharmacokinetics population)

	All patients		ADA-positive patients		ADA-negative patients	
	PF-06438179/GP1111	Infliximab-EU	PF-06438179/GP1111	Infliximab-EU	PF-06438179/GP1111	Infliximab-EU
C_trough_, median (5th–95th percentile), ng/ml						
Week 0 (day 1)	*n* = 322 0 (0–0)	*n* = 323 0 (0–0)	*n* = 156 0 (0–0)	*n* = 166 0 (0–0)	*n* = 163 0 (0–0)	*n* = 156 0 (0–0)
Week 2	*n* = 316 16,830 (6241–28,660)	*n* = 323 16,070 (6241–27,270)	*n* = 155 15,540 (5675–26,780)	*n* = 166 14,230 (5243–26,130)	*n* = 161 18,230 (6316–28,830)	*n* = 157 18,020 (9075–29,630)
Week 4	*n* = 308 23,540 (4300–45,750)	*n* = 314 21,250 (2258–40,120)	*n* = 151 17,760 (765–37,420)	*n* = 164 16,370 (256–32,450)	*n* = 157 27,850 (10,660–49,180)	*n* = 150 26,880 (12,980–41,390)
Week 6	*n* = 308 10,020 (102–26,650)	*n* = 315 9266 (0–24,180)	*n* = 151 6159 (0–20,180)	*n* = 163 5122 (0–17,440)	*n* = 157 14,030 (3960–29,890)	*n* = 152 12,790 (4321–26,420)
Week 14	*n* = 302 1497 (0–10,590)	*n* = 310 1025 (0–7643)	*n* = 154 0 (0–4014)	*n* = 159 0 (0–3428)	*n* = 148 3351 (492–15,660)	*n* = 151 3063 (197–8440)
Week 22	*n* = 295 576 (0–7911)	*n* = 303 433 (0–6221)	*n* = 152 0 (0–2262)	*n* = 156 0 (0–1151)	*n* = 143 2977 (206–10,640)	*n* = 147 2489 (0–7577)
Week 30	*n* = 281 413 (0–7253)	*n* = 290 279 (0–6017)	*n* = 143 0 (0–533)	*n* = 149 0 (0–575)	*n* = 138 2846 (386–10,050)	*n* = 141 2385 (192–7580)
C_max_, median (5th–95th percentile), ng/ml						
Week 0 (day 1)	*n* = 319 64,240 (31,570–102,000)	*n* = 322 62,200 (23,260–95,990)	*n* = 154 63,830 (35,630–101,500)	*n* = 166 59,290 (1603–93,170)	*n* = 162 65,530 (11,180–102,000)	*n* = 155 66,080 (29,140–101,200)
Week 14	*n* = 297 71,250 (1617–150,500)	*n* = 299 68,450 (3367–144,500)	*n* = 149 68,280 (0–157,500)	*n* = 152 62,010 (1091–118,200)	*n* = 148 75,640 (5633–129,400)	*n* = 147 75,090 (8857–159,800)

*Abbreviations: ADA* Antidrug antibody, *C*_*max*_ Observed serum drug concentration prior to the end of infusion, *C*_*trough*_ Observed predose trough serum concentration, *Infliximab-EU* Infliximab sourced from the European Union

## Discussion

The availability of biosimilars may expand access to biologic therapies such as infliximab, providing patients with additional safe and efficacious treatment options. Regulatory approval for biosimilars relies on a demonstration of biosimilarity that establishes there are no clinically meaningful differences in safety, purity, or potency between the proposed biosimilar and the originator product [8]. This determination of biosimilarity is made on the basis of the totality of the evidence obtained from all stages of the development process, which begins with comprehensive analytical (i.e., structural and functional) characterization followed by nonclinical testing [7, 8]. Finally, a confirmatory clinical study (or studies) is conducted to demonstrate similarity between the proposed biosimilar and the originator product in terms of their PK, efficacy, safety, and immunogenicity profiles [7, 8].

As the final step in the biosimilarity exercise, this study was conducted to compare the efficacy and safety of PF-06438179/GP1111 and infliximab-EU. The study met its primary objective by demonstrating therapeutic equivalence between PF-06438179/GP1111 and infliximab-EU. The 95% and 90% CIs for the difference between arms in week 14 ACR20 response rates were entirely contained within the prespecified symmetric and asymmetric equivalence margins, respectively. Compared with the symmetric margin, the asymmetric margin applied a smaller lower bound but a larger upper bound for the CI. Although this is less stringent for higher efficacy of the biosimilar, it is more stringent for potential lower efficacy of the biosimilar. Sensitivity analyses confirmed that the comparison of PF-06438179/GP1111 and infliximab-EU in ACR20 response rate at week 14 was robust under different missing data imputation approaches. No differences were observed between PF-06438179/GP1111 and infliximab-EU for secondary efficacy endpoints, supporting the results of the primary endpoint analysis.

The week 14 ACR20 responses observed in this study (62.7% for PF-06438179/GP1111 and 64.1% for infliximab-EU) were within the range of ACR20 response rates reported in historical registration trials for infliximab (Remicade®; 50–76%) [21, 22, 24, 25]. Furthermore, the current study and historical reference trials for infliximab were generally similar in terms of patient enrollment criteria, MTX dosing, and patient demographic and baseline disease characteristics [21, 22, 24, 25]. The primary assessment of ACR20 response was evaluated at week 14, an earlier time point than used in the historical reference studies of infliximab vs placebo in patients with RA [3, 4, 22–25]. Evaluation of ACR20 response early in the therapeutic plateau provides greater sensitivity to detect possible differences in the rate of response between treatment arms, as compared with later time points. This is relevant and appropriate for biosimilarity studies, which are designed to demonstrate there are no clinically meaningful differences between treatments [8, 20].

The safety profiles of PF-06438179/GP1111 and infliximab-EU were similar, with no clinically meaningful differences observed between arms. The incidence and characteristics of TEAEs of special interest were similar between treatment arms, including IRRs and hypersensitivity events for both arms and for the ADA-positive subsets following ADA onset. The incidence of patients with at least one positive postdose ADA result during the 30-week treatment period (48.6% for PF-06438179/GP1111 and 51.2% for infliximab-EU) and the percentage of ADA-positive patients who tested positive for NAb (79.0% for PF-06438179/GP1111 and 85.6% for infliximab-EU) were similar between the two arms. Incidence of ADAs in both arms was higher than reported in a pivotal infliximab trial (~ 22%) [24]; however, this is attributed to the higher sensitivity of electrochemiluminescence assays, which result in detection of lower-titer ADA. Evaluation of immunogenicity during the initial 30-week treatment period was supported by the higher sensitivity of current methods to detect the development of ADA. Serum PF-06438179/GP1111 and infliximab-EU concentrations were similar at each time point during the first 30 weeks of dosing.

The current study incorporated dose escalation to 5 mg/kg infliximab, starting at week 14, for patients with an inadequate RA response. Efficacy and immunogenicity profiles were comparable between PF-06438179/GP1111 and infliximab-EU for patients with dose escalation to 5 mg/kg, as well as between the two treatment arms for patients without dose escalation. These data are reassuring because dose optimization is common for infliximab and because patients with RA who experience nonresponse, inadequate response, or loss of response generally demonstrate improvement after a dose increase [27–29]. Furthermore, the placebo-adjusted response to infliximab in RA is greater with higher doses of infliximab [3, 4, 30], which increases the sensitivity of RA as a clinical model for detecting potential differences between originator infliximab and proposed infliximab biosimilars [30]. Therefore, evidence for similar efficacy between PF-06438179/GP1111 and infliximab-EU at the 5 mg/kg dose could support its use in other indications (e.g., inflammatory bowel disease, ankylosing spondylitis) for which infliximab is approved.

## Conclusions

PF-06438179/GP1111 and infliximab-EU demonstrated similar efficacy, safety, immunogenicity, and PK profiles in patients with moderate to severe active RA on background MTX up to 30 weeks. Furthermore, efficacy and immunogenicity of PF-06438179/GP1111 and infliximab-EU were comparable for patients with dose escalation to 5 mg/kg infliximab, as well as between arms for patients without dose escalation. The results of this study, combined with previous results of an analytical (structural and functional) evaluation [15], demonstrate similarity of PF-06438179/GP1111 to infliximab-EU. This trial will also evaluate clinical efficacy, safety, and immunogenicity after a single transition from infliximab-EU to PF-06438179/GP1111 after 30 or 54 weeks of treatment.

## Additional file

Additional file 1: Supplementary methods and results, supplementary figures and figure legends, and supplementary tables. (PDF 394 kb)

## Abbreviations

ACR: American College of Rheumatology
ACR20/50/70: American College of Rheumatology criteria for ≥ 20%/50%/70% clinical improvement
ADA: Antidrug antibody
AE: Adverse event
Anti-CCP: Anticyclic citrullinated peptide
C_max_: Observed serum drug concentration prior to the end of infusion
C_trough_: Observed predose trough serum concentration
DAS: Disease Activity Score
DAS28-CRP: Disease Activity Score in 28 joints, four components based on C-reactive protein
DMARD: Disease-modifying antirheumatic drugs
EOT: End of treatment
EU: European Union
EULAR: European League Against Rheumatism
HAQ-DI: Health Assessment Questionnaire Disability Index
hs-CRP: High-sensitivity C-reactive protein
Infliximab-EU/US: Infliximab sourced from the European Union/United States
IRR: Infusion-related reaction
ITT: Intention to treat
MedDRA: Medical Dictionary for Regulatory Activities
MTX: Methotrexate
NAb: Neutralizing antibody
NRI: Nonresponder imputation
PD: Pharmacodynamics
PK: Pharmacokinetics
PP: Per protocol
RA: Rheumatoid arthritis
RF: Rheumatoid factor
SAE: Serious adverse event
SOC: System Organ Class
TEAE: Treatment-emergent adverse event
TNF: Tumor necrosis factor

## References

[1] European Medicines Agency (EMA) (2017). Remicade (infliximab) summary of product characteristics. London: EMA.

[2] Janssen Biotech Inc. Remicade (infliximab) US prescribing information. Horsham, PA: Janssen Biotech, Inc.; 2017. https://www.accessdata.fda.gov/drugsatfda_docs/label/2017/761072s000lbl.pdf. Accessed 14 Aug 2017.

[3] Lipsky PE, van der Heijde DM, St Clair EW, Furst DE, Breedveld FC, Kalden JR (2000). Infliximab and methotrexate in the treatment of rheumatoid arthritis: anti-tumor necrosis factor trial in rheumatoid arthritis with concomitant therapy study group. N Engl J Med.

[4] St Clair EW, van der Heijde DM, Smolen JS, Maini RN, Bathon JM, Emery P (2004). Combination of infliximab and methotrexate therapy for early rheumatoid arthritis: a randomized, controlled trial. Arthritis Rheum.

[5] Kalo Z, Voko Z, Ostor A, Clifton-Brown E, Vasilescu R, Battersby A (2017). Patient access to reimbursed biological disease-modifying antirheumatic drugs in the European region. J Mark Access Health Policy.

[6] Cifaldi M, Renaud J, Ganguli A, Halpern MT (2016). Disparities in care by insurance status for individuals with rheumatoid arthritis: analysis of the medical expenditure panel survey, 2006-2009. Curr Med Res Opin.

[7] Committee for Medicinal Products for Human Use (CHMP), European Medicines Agency (EMA). Guideline on similar biological medicinal products. London: EMA; 2014. http://www.ema.europa.eu/docs/en_GB/document_library/Scientific_guideline/2014/10/WC500176768.pdf. Accessed 14 Aug 2017.

[8] US Food and Drug Administration (FDA) (2015). Scientific considerations in demonstrating biosimilarity to a reference product. Guidance for industry. Silver Spring, MD: FDA.

[9] IMS Institute for Healthcare Informatics. Delivering on the potential of biosimilar medicines: the role of functioning competitive markets. Parsippany, NJ: IMS Health and the IMS Institute for Healthcare Informatics; March 2016. https://www.medicinesforeurope.com/wp-content/uploads/2016/03/IMS-Institute-Biosimilar-Report-March-2016-FINAL.pdf. Accessed 21 Aug 2017.

[10] Jha A, Upton A, Dunlop WC, Akehurst R (2015). The budget impact of biosimilar infliximab (Remsima®) for the treatment of autoimmune diseases in five European countries. Adv Ther.

[11] Singh SC, Bagnato KM (2015). The economic implications of biosimilars. Am J Manag Care.

[12] Jorgensen KK, Olsen IC, Goll GL, Lorentzen M, Bolstad N, Haavardsholm EA (2017). Switching from originator infliximab to biosimilar CT-P13 compared with maintained treatment with originator infliximab (NOR-SWITCH): a 52-week, randomised, double-blind, non-inferiority trial. Lancet.

[13] Pfizer Inc. Ixifi (infliximab-qbtx) US prescribing information. Pfizer Inc. 2017. https://www.accessdata.fda.gov/drugsatfda_docs/label/2017/761072s000lbl.pdf. Accessed 2 July 2018.

[14] European Medicines Agency. Zessly (infliximab) summary of product characteristics. 2018. http://www.ema.europa.eu/docs/en_GB/document_library/EPAR_-_Product_Information/human/004647/WC500249647.pdf. Accessed 2 July 2018.

[15] Derzi M, Johnson TR, Shoieb AM, Conlon HD, Sharpe P, Saati A (2016). Nonclinical evaluation of PF-06438179: a potential biosimilar to Remicade® (infliximab). Adv Ther.

[16] Palaparthy R, Udata C, Hua SY, Yin D, Cai CH, Salts S (2018). A randomized study comparing the pharmacokinetics of the potential biosimilar PF-06438179/GP1111 with Remicade® (infliximab) in healthy subjects (REFLECTIONS B537-01). Expert Rev Clin Immunol.

[17] Aletaha D, Neogi T, Silman AJ, Funovits J, Felson DT, Bingham CO (2010). 2010 rheumatoid arthritis classification criteria: an American College of Rheumatology/European League Against Rheumatism collaborative initiative. Ann Rheum Dis.

[18] Hochberg MC, Chang RW, Dwosh I, Lindsey S, Pincus T, Wolfe F (1992). The American College of Rheumatology 1991 revised criteria for the classification of global functional status in rheumatoid arthritis. Arthritis Rheum.

[19] ClinicalTrials.gov. A study of PF-06438179 (infliximab-Pfizer) and infliximab in combination with methotrexate in subjects with active rheumatoid arthritis (REFLECTIONS B537–02). Registered on 21 Aug 2014. https://clinicaltrials.gov/ct2/show/NCT02222493. Accessed 14 Aug 2017.

[20] US Food and Drug Administration (FDA) (2013). Guidance for industry. Rheumatoid arthritis: developing drug products for treatment. Rockville, MD: U.S. Department of Health and Human Services, FDA, Center for Drug Evaluation and Research (CDER).

[21] Abe T, Takeuchi T, Miyasaka N, Hashimoto H, Kondo H, Ichikawa Y (2006). A multicenter, double-blind, randomized, placebo controlled trial of infliximab combined with low dose methotrexate in Japanese patients with rheumatoid arthritis. J Rheumatol.

[22] Maini R, St Clair EW, Breedveld F, Furst D, Kalden J, Weisman M (1999). Infliximab (chimeric anti-tumour necrosis factor α monoclonal antibody) versus placebo in rheumatoid arthritis patients receiving concomitant methotrexate: a randomised phase III trial. ATTRACT study group. Lancet.

[23] Schiff M, Keiserman M, Codding C, Songcharoen S, Berman A, Nayiager S (2008). Efficacy and safety of abatacept or infliximab vs placebo in ATTEST: a phase III, multi-centre, randomised, double-blind, placebo-controlled study in patients with rheumatoid arthritis and an inadequate response to methotrexate. Ann Rheum Dis.

[24] Westhovens R, Yocum D, Han J, Berman A, Strusberg I, Geusens P (2006). The safety of infliximab, combined with background treatments, among patients with rheumatoid arthritis and various comorbidities: a large, randomized, placebo-controlled trial. Arthritis Rheum.

[25] Zhang F-C, Hou Y, Huang F, Wu D-H, Bao C-D, Ni L-Q (2006). Infliximab versus placebo in rheumatoid arthritis patients receiving concomitant methotrexate: a preliminary study from China. APLAR J Rheumatol.

[26] Hua SY, Xu S, Barker KB, Liao SM, Li S, Barker KB, Menon SM, D’ Agostino RB, Xu S, Jin B (2017). Bayesian methods to assess bioequivalence and biosimilarity with case studies. Biosimilar clinical development: scientific considerations and new methodologies.

[27] Alten R, van den Bosch F (2014). Dose optimization of infliximab in patients with rheumatoid arthritis. Int J Rheum Dis.

[28] Ariza-Ariza R, Navarro-Sarabia F, Hernandez-Cruz B, Rodriguez-Arboleya L, Navarro-Compan V, Toyos J (2007). Dose escalation of the anti-TNF-α agents in patients with rheumatoid arthritis: a systematic review. Rheumatology (Oxford).

[29] Wu E, Chen L, Birnbaum H, Yang E, Cifaldi M (2008). Retrospective claims data analysis of dosage adjustment patterns of TNF antagonists among patients with rheumatoid arthritis. Curr Med Res Opin.

[30] Lee H (2014). Is extrapolation of the safety and efficacy data in one indication to another appropriate for biosimilars?. AAPS J.

