# Supplementary material for: A randomized controlled trial comparing PF-06438179/GP1111 (an infliximab biosimilar) and infliximab reference product for treatment of moderate to severe active rheumatoid arthritis despite methotrexate therapy
Source: Arthritis Res Ther. 2018 Jul 27;20:155. doi: 10.1186/s13075-018-1646-4 (PMC6063022; doi:10.1186/s13075-018-1646-4)
Supplement: Supplementary file 1 — Supplementary methods and results, supplementary figures and figure legends, and supplementary tables. (PDF 394 kb) [file 13075_2018_1646_MOESM1_ESM.pdf]

A randomized controlled trial comparing PF-06438179/GP1111 (an infliximab biosimilar) and infliximab reference product for treatment of moderate to severe active rheumatoid arthritis despite methotrexate therapy

Stanley B. Cohen[1]*, Rieke Alten[2], Hideto Kameda[3], Tomas Hala[4], Sebastiao C. Radominski[5], Muhammad I. Rehman[6], Ramesh Palaparthy[7], Karl Schumacher[8], Susanne Schmitt[8], Steven Y. Hua[7], Claudia Ianos[9], K. Lea Sewell[10]

[1]Metroplex Clinical Research Center, 8144 Walnut Hill Lane, Suite 810, Dallas, TX 75231, USA; [2]Schlosspark-Klinik University Medicine, Heubnerweg 2, 14059 Berlin, Germany; [3]Toho University Ohashi Medical Center, 2-17-6, Ohashi Muguro-ku, Tokyo 153-8515, Japan; [4]Center for Clinical and Basic Research, Trida Miru 2800, 530 02, Pardubice, Czech Republic; [5]Universidade Federal do Paraná, Rua General Carneiro, 181 - Alto da Glória, Curitiba, PR 80.060-900, Brazil; [6]Pfizer Inc., 1 Burtt Road, Andover, MA 01810, USA; [7]Pfizer Inc., 10777 Science Center Drive, CB1/2103, San Diego, CA 92121, USA; [8]Hexal AG, Industriestraße 25, D-83607 Holzkirchen, Germany; [9]Pfizer UK., Discovery Park, Ramsgate Road, Sandwich CT13 9ND, UK; [10]Pfizer Inc., 300 Technology Square, Cambridge, MA 02139, USA

* Correspondence: arthdoc@aol.com; Metroplex Clinical Research Center, 8144 Walnut Hill Lane, Suite 810, Dallas, TX 75231, USA

Additional file 1

Supplementary Statistical Methods

The tipping point analysis for ACR20 at week 14 was performed for the asymmetric margin using a sequential logistic regression imputation model that included terms for region, ACR20 at weeks 6 and 12 (both potentially imputed), and treatment. This analysis varied the assumed probabilities of response among patients with missing data via a series of delta adjustments over the range of –1 to +1 that increased the treatment difference relative to logistic regression imputation without adjustment (delta=0). Tipping points were defined by deltas associated with a 90% CI that was not entirely contained within the asymmetric equivalence margin (–12%, 15%).

Supplementary Results

Sensitivity analyses for ACR20 at week 14

In a sensitivity analysis of the ITT population that incorporated one additional responder in the infliximab-EU arm who was identified at site closeout, 203 (62.7%) and 210 (64.4%) patients in the PF-06438179/GP1111 and infliximab-EU arms, respectively, achieved ACR20 response at week 14. Using NRI, 198 (61.1%) and 208 (63.8%) patients in the PF-06438179/GP1111 and infliximab-EU arms, respectively, had a week 14 ACR20 response. The 95% (–10.21%, 4.80%) and 90% (–9.05%, 3.62%) CIs for the treatment difference (–2.69%) were entirely contained within the pre-specified symmetric and asymmetric equivalence margins, respectively (Supplementary Figure S2). ACR20 responses at week 14 for the PP population were similar to those reported for the ITT population for both PF-06438179/GP1111 (186/279 [66.7%]) and infliximab-

EU (196/290 [67.6%]). Furthermore, the 95% (–8.77, 6.87) and 90% (–7.50, 5.66) CIs for the treatment difference (–0.92) were entirely contained within the pre-specified symmetric and asymmetric equivalence margins, respectively (Supplementary Figure S2).

In the analysis using observed data (without NRI), the 95% and 90% CIs for the treatment difference in ACR20 response at week 14 were all contained within the pre-specified equivalence margins. Likewise, the 95% and 90% CIs for the treatment difference in ACR20 response at week 14 were contained within the pre-specified equivalence margins in the analysis adjusting for geographic region (Supplementary Table S1). Furthermore, a tipping point analysis based on multiple imputation demonstrated the asymmetric equivalence criterion was uniformly met over the range of adjustments (delta of –1 to +1) for treatment difference that were explored.

ACR20 responses over time

CR20 response rates across all study visits up to week 30 were analyzed using a generalized linear model for repeated-measure data, adjusting for geographic region. Although the lower bounds of the 95% and 90% CIs for the week 4 ACR20 response rate treatment difference in the PP population were slightly outside of the symmetric and asymmetric margins, respectively, which were only defined for week 14 for the primary endpoint, results in Supplementary Table S2 show that the equivalence criteria were achieved at all other study visits (i.e., at 6 of the 7 post-treatment visits). This finding in and of itself provided clear evidence for similarity between the two treatment groups.

Supplementary Tables

Supplementary Table S1 Sensitivity analysis of ACR20 response at week 14, adjusting for geographic region[a]

	**PF-06438179/GP1111**	**Infliximab-EU**
ITT population		
Patients with non-missing data, *N*	311	316
Patients with ACR20 response, *n* (%)	203 (65.3)	209 (66.1)
Difference between treatments in ACR20 response (SE) [95% CI]	–1.51 (3.74) [–8.83, 5.82]	
Difference between treatments in ACR20 response (SE) [90% CI]	–1.51 (3.74) [–7.65, 4.64]	
PP population		
Patients with non-missing data, *N*	279	290
Patients with ACR20 response, *n* (%)	186 (66.7)	195 (67.2)
Difference between treatments in ACR20 response (SE) [95% CI]	–1.39 (3.89) [–9.01, 6.23]	
Difference between treatments in ACR20 response (SE) [90% CI]	–1.39 (3.89) [–7.79, 5.01]	

[a] Analysis was performed using observed data and included the fixed effect of randomized treatment and covariate of geographic region.

ACR20, American College of Rheumatology criteria for ≥20% clinical improvement; CI, confidence interval; infliximab-EU, infliximab sourced from the European Union; ITT, intention to treat; PP, per-protocol; SE, standard error.

Supplementary Table S2 Repeated measures analysis of ACR20 response rate up to week 30, adjusting for geographic region[a]

Visit	PF-06438179/GP1111, *n/N* (%)	Infliximab-EU, *n/N* (%)	Difference between treatments in ACR20 response (SE)	95% CI	90% CI
ITT population					
Week 2	105/319 (32.9)	121/324 (37.4)	–4.77 (3.78)	–12.19, 2.64	–10.99, 1.45
Week 4	170/317 (53.6)	190/321 (59.2)	–5.72 (3.91)	–13.37, 1.93	–12.14, 0.70
Week 6	187/313 (59.7)	201/319 (63.0)	–3.85 (3.83)	–11.34, 3.65	–10.14, 2.45
Week 12	210/311 (67.5)	214/318 (67.3)	0.15 (3.72)	–7.14, 7.43	–5.97, 6.26
Week 14	203/311 (65.3)	209/316 (66.1)	–1.14 (3.75)	–8.50, 6.21	–7.32, 5.03
Week 22	205/301 (68.1)	213/311 (68.5)	–0.04 (3.72)	–7.33, 7.26	–6.16, 6.08
Week 30	197/294 (67.0)	209/298 (70.1)	–2.28 (3.78)	–9.69, 5.13	–8.50, 3.94
PP population					
Week 2	93/278 (33.5)	109/290 (37.6)	–4.52 (4.04)	–12.44, 3.40	–11.16, 2.13
Week 4	151/277 (54.5)	175/287 (61.0)	–6.47 (4.15)	–14.60, 1.66	–13.30, 0.35

Week 6	168/278 (60.4)	184/289 (63.7)	–3.95 (4.03)	–11.85, 3.95	–10.58, 2.68
Week 12	191/278 (68.7)	199/288 (69.1)	–0.85 (3.90)	–8.48, 6.79	–7.26, 5.56
Week 14	186/279 (66.7)	195/290 (67.2)	–1.22 (3.91)	–8.88, 6.45	–7.65, 5.22
Week 22	189/276 (68.5)	200/285 (70.2)	–1.27 (3.83)	–8.78, 6.24	–7.57, 5.03
Week 30	186/269 (69.1)	197/273 (72.2)	–2.08 (3.86)	–9.64, 5.48	–8.43, 4.26

[a] Analysis included the fixed effects of randomized treatment, visit, and treatment-by-visit interaction and the covariate of geographic region. ACR20 response rate was calculated based on the number of patients with ACR20 response/the number of patients with non-missing ACR20 response data at each study visit.

ACR20, American College of Rheumatology criteria for ≥20% clinical improvement; CI, confidence interval; infliximab-EU, infliximab sourced from the European Union; ITT, intention to treat; PP, per-protocol; SE, standard error.

Supplementary Table S3 Rates of EULAR response by study visit (ITT population)

	Week 2, *n* (%)	Week 4, *n* (%)	Week 6, *n* (%)	Week 12, *n* (%)	Week 14, *n* (%)	Week 22, *n* (%)	Week 30, *n* (%)
PF-06438179/GP1111 (*n* = 324)							
EULAR response							
Good	24 (7.4)	61 (18.8)	65 (20.1)	90 (27.8)	97 (29.9)	103 (31.8)	101 (31.2)
Moderate	172 (53.1)	162 (50.0)	168 (51.9)	149 (46.0)	137 (42.3)	125 (38.6)	133 (41.1)
None	121 (37.4)	89 (27.5)	79 (24.4)	71 (21.9)	76 (23.5)	73 (22.5)	58 (17.9)
Infliximab-EU (*n* = 326)							
EULAR response							
Good	34 (10.4)	56 (17.2)	64 (19.6)	88 (27.0)	82 (25.2)	96 (29.5)	94 (28.8)
Moderate	161 (49.4)	172 (52.8)	181 (55.5)	162 (49.7)	155 (47.6)	156 (47.9)	155 (47.6)
None	129 (39.6)	87 (26.7)	74 (22.7)	66 (20.3)	77 (23.6)	55 (16.9)	48 (14.7)

EULAR, European League Against Rheumatism; infliximab-EU, infliximab sourced from the European Union; ITT, intention to treat.

Supplementary Table S4 Rates of DAS and ACR/EULAR remission by study visit (ITT population)

	Week 2, *n* (%)	Week 4, *n* (%)	Week 6, *n* (%)	Week 12, *n* (%)	Week 14, *n* (%)	Week 22, *n* (%)	Week 30, *n* (%)
PF-06438179/GP1111 (*n* = 324)							
DAS remission							
Yes	9 (2.8)	28 (8.6)	40 (12.4)	52 (16.1)	53 (16.4)	58 (17.9)	62 (19.1)
No	311 (96.0)	289 (89.2)	274 (84.6)	260 (80.3)	258 (79.6)	243 (75.0)	232 (71.6)
ACR/EULAR remission							
Yes	2 (0.6)	10 (3.1)	12 (3.7)	28 (8.6)	27 (8.3)	25 (7.7)	30 (9.3)
No	318 (98.2)	307 (94.8)	302 (93.2)	284 (87.7)	284 (87.7)	276 (85.2)	264 (81.5)
Infliximab-EU (*n* = 326)							
DAS remission							
Yes	17 (5.2)	32 (9.8)	35 (10.7)	44 (13.5)	43 (13.2)	50 (15.3)	54 (16.6)
No	307 (94.2)	289 (88.7)	285 (87.4)	274 (84.1)	273 (83.7)	261 (80.1)	244 (74.9)
ACR/EULAR remission							
Yes	3 (0.9)	11 (3.4)	10 (3.1)	17 (5.2)	22 (6.8)	20 (6.1)	23 (7.1)

No	321 (98.5)	310 (95.1)	310 (95.1)	301 (92.3)	294 (90.2)	291 (89.3)	275 (84.4)

ACR, American College of Rheumatology; DAS, Disease Activity Score; EULAR, European League Against Rheumatism; infliximab-EU, infliximab sourced from the European Union; ITT, intention to treat.

Supplementary Table S5 All-causality TEAEs of special interest (safety population)

**Event of special interest, *n* (%)**	**PF-06438179/GP1111 (*n* = 323)**	**Infliximab-EU (*n* = 326)**
IRR	19 (5.9)	21 (6.4)
Hypersensitivity	44 (13.6)	51 (15.6)
Infections	87 (26.9)	73 (22.4)
Tuberculosis	1 (0.3)	1 (0.3)
Active	0	1 (0.3)
Latent	1 (0.3)	0
Pneumonia	3 (0.9)	3 (0.9)
Pneumonia	2 (0.6)	3 (0.9)
Pneumocystis jirovecii pneumonia	1 (0.3)	0
Neoplasms	1 (0.3)	2 (0.6)
Malignancies (colon cancer)	1 (0.3)	1 (0.3)

IRR, infusion-related reactions; infliximab-EU, infliximab sourced from the European Union; TEAE, treatment-emergent adverse event.

Supplementary Table S6 ADA and NAb incidence by study visit (safety population)

Study visit	ADA and NAb status[a]	PF-06438179/GP1111 (*n* = 323), *n* (%)	Infliximab-EU (*n* = 326), *n* (%)
Week 0 (Baseline)	ADA positive	9 (2.8)	9 (2.8)
	NAb positive	1 (11.1)	1 (11.1)
	NAb negative	8 (88.9)	8 (88.9)
	ADA negative	313 (96.9)	314 (96.3)
	ADA not done	1 (0.3)	3 (0.9)
Week 2	ADA positive	10 (3.1)	8 (2.5)
	NAb positive	3 (30.0)	3 (37.5)
	NAb negative	7 (70.0)	5 (62.5)
	ADA negative	308 (95.4)	315 (96.6)
	ADA not done	5 (1.5)	3 (0.9)
Week 6	ADA positive	22 (6.8)	24 (7.4)
	NAb positive	13 (59.1)	19 (79.2)
	NAb negative	8 (36.4)	5 (20.8)
	ADA negative	285 (88.2)	293 (89.9)
	ADA not done	16 (5.0)	9 (2.8)
Week 14	ADA positive	96 (29.7)	100 (30.7)
	NAb positive	73 (76.0)	78 (78.0)
	NAb negative	23 (24.0)	22 (22.0)

	ADA negative	206 (63.8)	214 (65.6)
	ADA not done	21 (6.5)	12 (3.7)
Week 30	ADA positive	136 (42.1)	144 (44.2)
	NAb positive	105 (77.2)	120 (83.3)
	NAb negative	31 (22.8)	23 (16.0)
	ADA negative	146 (45.2)	147 (45.1)
	ADA not done	41 (12.7)	35 (10.7)
Overall	ADA positive	157 (48.6)	167 (51.2)
	NAb positive	124 (79.0)	143 (85.6)
	NAb negative	33 (21.0)	23 (13.8)
	ADA negative	163 (50.5)	158 (48.5)
	ADA not done	3 (0.9)	1 (0.3)

[a] ADA-positive and negative test results were defined as ADA titer ≥ 1.30 and < 1.30, respectively. Overall, a patient who tested positive was defined as having ≥ 1 postdose positive sample during the 30-week treatment period, regardless of predose ADA status. ADA not done includes samples that were not collected or that were collected but not analyzed. NAb-positive and negative results were defined as NAb titer ≥ 0.70 and < 0.70, respectively. Incidences of NAb-positive and negative patients are expressed as percentages of ADA-positive patients.

ADA, anti-drug antibody; infliximab-EU, infliximab sourced from the European Union; NAb, neutralizing antibody.

Supplementary Table S7 ADA and NAb incidence by study visit for patients who dose escalated at week 14 or 22 (safety population)[a]

	No dose-escalation (3 mg/kg)		Dose-escalation (5 mg/kg)	
	PF-06438179/ GP1111 (*n* = 240), *n* (%)	Infliximab-EU (*n* = 243), *n* (%)	PF-06438179/ GP1111 (*n* = 83), *n* (%)	Infliximab-EU (*n* = 83), *n* (%)
Week 14 (predose)				
ADA positive[b]	64 (26.7)	63 (25.9)	32 (38.6)	37 (44.6)
NAb positive[c]	50 (78.1)	47 (74.6)	23 (71.9)	31 (83.8)
NAb negative[c]	14 (21.9)	16 (25.4)	9 (28.1)	6 (16.2)
NAb not done[d]	0	0	0	0
ADA negative[b]	155 (64.6)	169 (69.5)	51 (61.4)	45 (54.2)
Not done[d]	21 (8.8)	11 (4.5)	0	1 (1.2)
Week 30 (predose)				
ADA positive[b]	98 (40.8)	112 (46.1)	38 (45.8)	32 (38.6)
NAb positive[c]	78 (79.6)	91 (81.3)	27 (71.1)	29 (90.6)
NAb negative[c]	20 (20.4)	20 (17.9)	11 (28.9)	3 (9.4)
NAb not done[d]	0	1 (0.9)	0	0
ADA negative[b]	112 (46.7)	111 (45.7)	34 (41.0)	36 (43.4)
Not done[d]	30 (12.5)	20 (8.2)	11 (13.3)	15 (18.1)

[a] ADA and NAb incidence at week 14 represent immunogenicity assessments done prior to any dose adjustment at either weeks 14 or 22; ADA and NAb incidence at week

30 represent immunogenicity assessments done after dose adjustment at either weeks 14 or 22, but prior to dose administration at week 30.

[b] ADA-positive and negative test results were defined as ADA titer ≥ 1.30 and < 1.30, respectively.

[c] NAb-positive and negative results were defined as NAb titer ≥ 0.70 and < 0.70, respectively. Incidences of NAb-positive patients are expressed as percentages of ADA-positive patients.

[d] Not done includes samples that were not collected and collected but not analyzed.

ADA, anti-drug antibody; infliximab-EU, infliximab sourced from the European Union; NAb, neutralizing antibody.

Supplementary Figure S1 Patient disposition

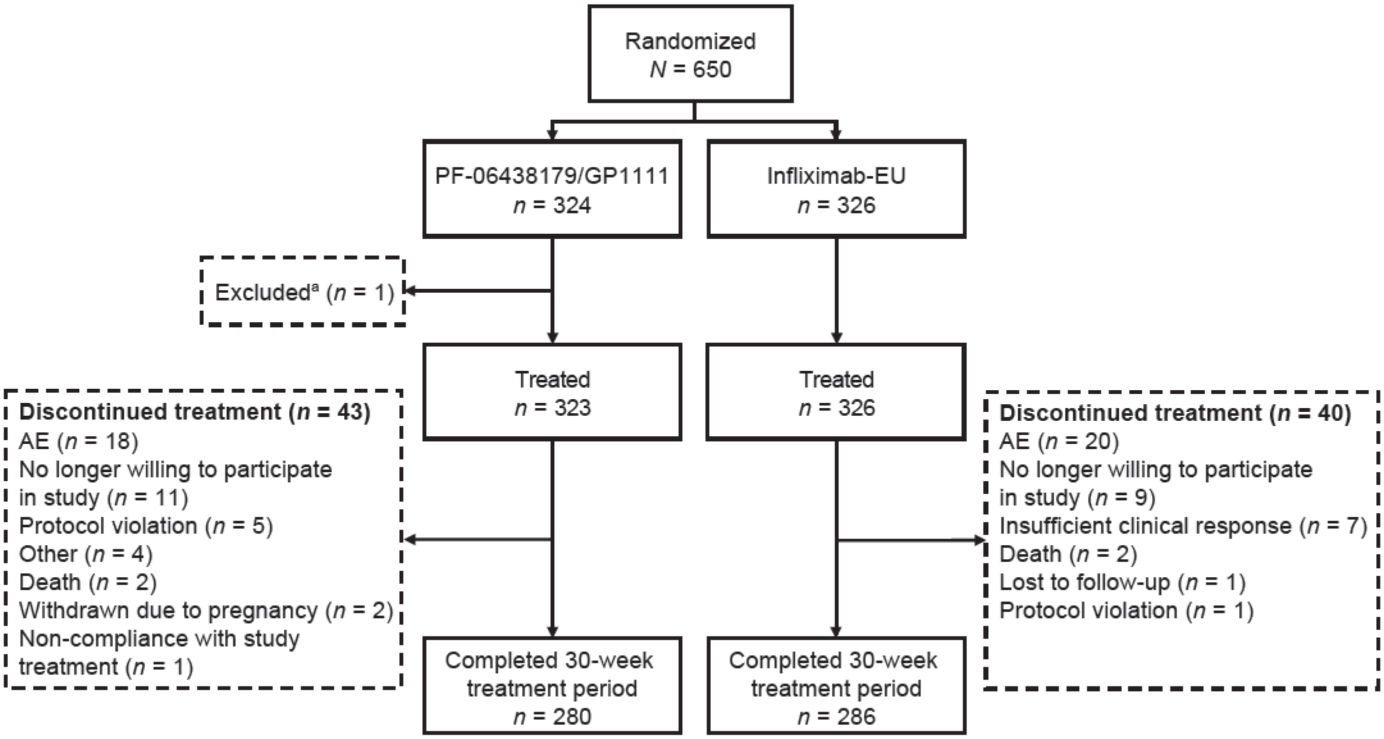

[a] One patient in the PF-06438179/GP1111 arm was randomized twice; data were not collected for this patient's second randomization.

AE, adverse event; infliximab-EU, infliximab sourced from the European Union.

Supplementary Figure S2 Sensitivity analysis of week 14 ACR20 response incorporating one additional responder in the infliximab-EU arm who was identified at site closeout

A) Difference (95% CI) in week 14 ACR20 response between PF-06438179/GP1111 and infliximab-EU using NRI and symmetric equivalence margin

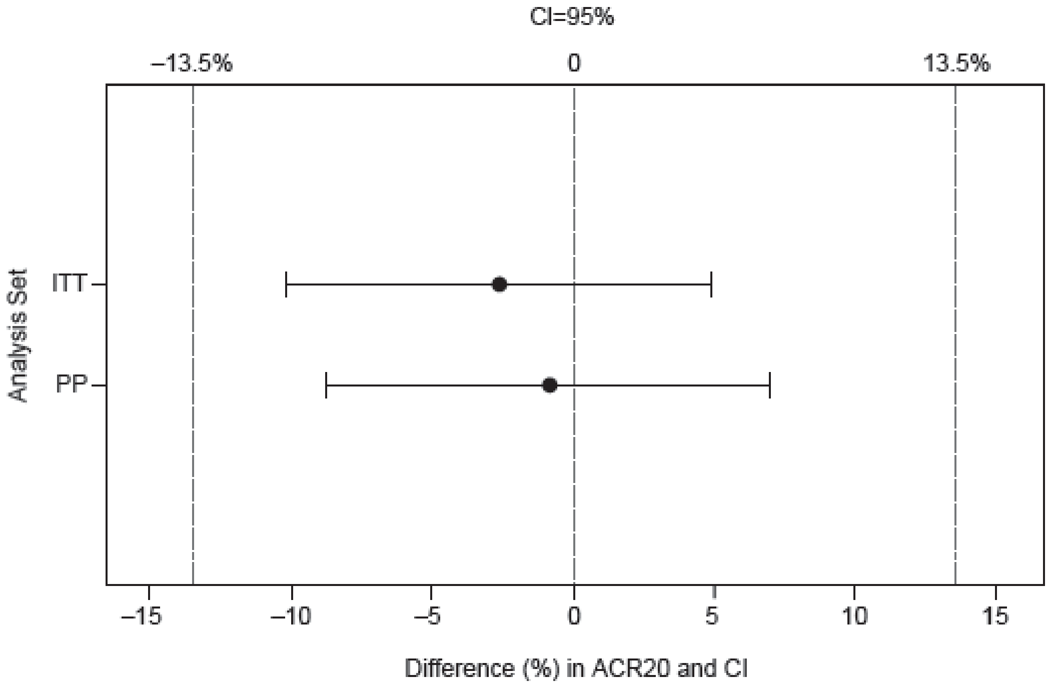

B) Difference (90% CI) in week 14 ACR20 response between PF-06438179/GP1111 and infliximab-EU using NRI and asymmetric equivalence margin

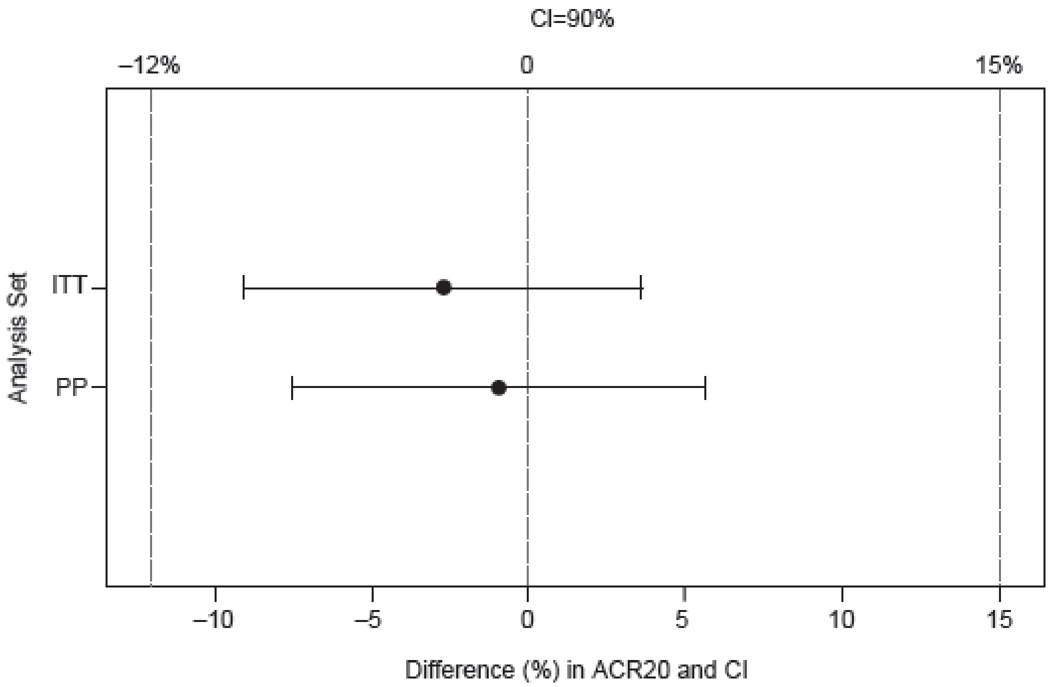

ACR20, American College of Rheumatology criteria for ≥20% clinical improvement; CI, confidence interval; infliximab-EU, infliximab sourced from the European Union; ITT, intention to treat; NRI, non-responder imputation; PP, per protocol.

Supplementary Figure S3 Mean (±SE) change from baseline in hs-CRP by visit (ITT population)

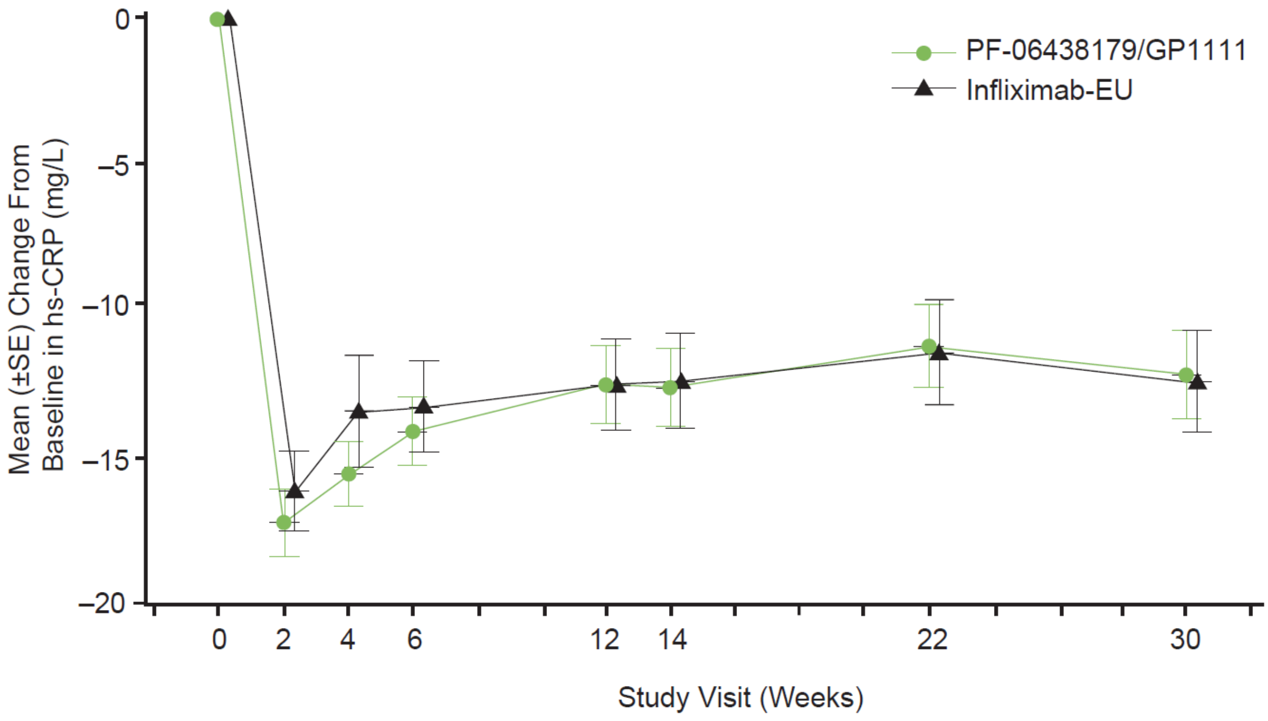

hs-CRP, high-sensitivity C-reactive protein; Infliximab-EU, infliximab sourced from the European Union; ITT, intention to treat; SE, standard error.

Supplementary Figure S4 ADA titers versus time for PF-06438179/GP1111 and infliximab-EU ADA-positive subgroups[a]

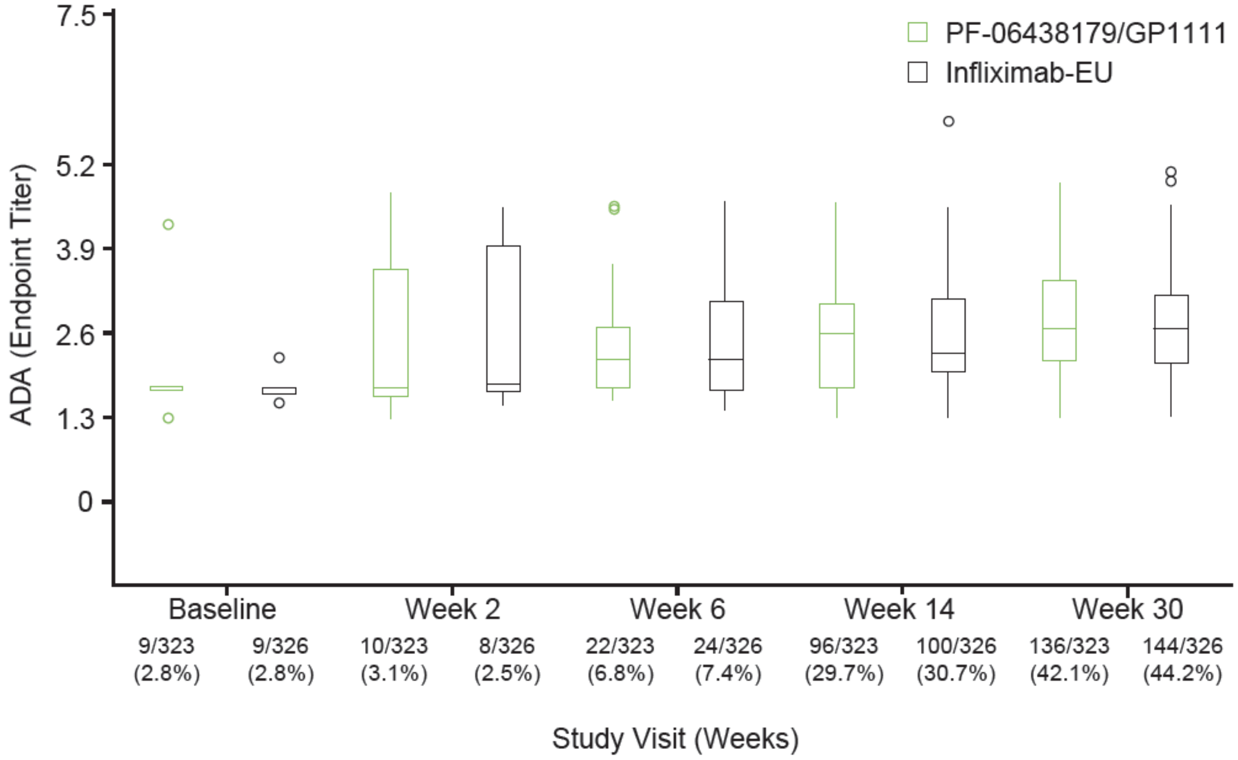

[a] ADA-positive test result was defined as ADA titer ≥ 1.3. Circles represent outliers. ADA incidence at each study visit are displayed along the bottom of the figure.

ADA, anti-drug antibody; infliximab-EU, infliximab sourced from the European Union.

Supplementary Figure S5 Serum drug concentration-time profile for PF-06438179/GP1111 and infliximab-EU ADA-positive subgroups (PK population)[a]

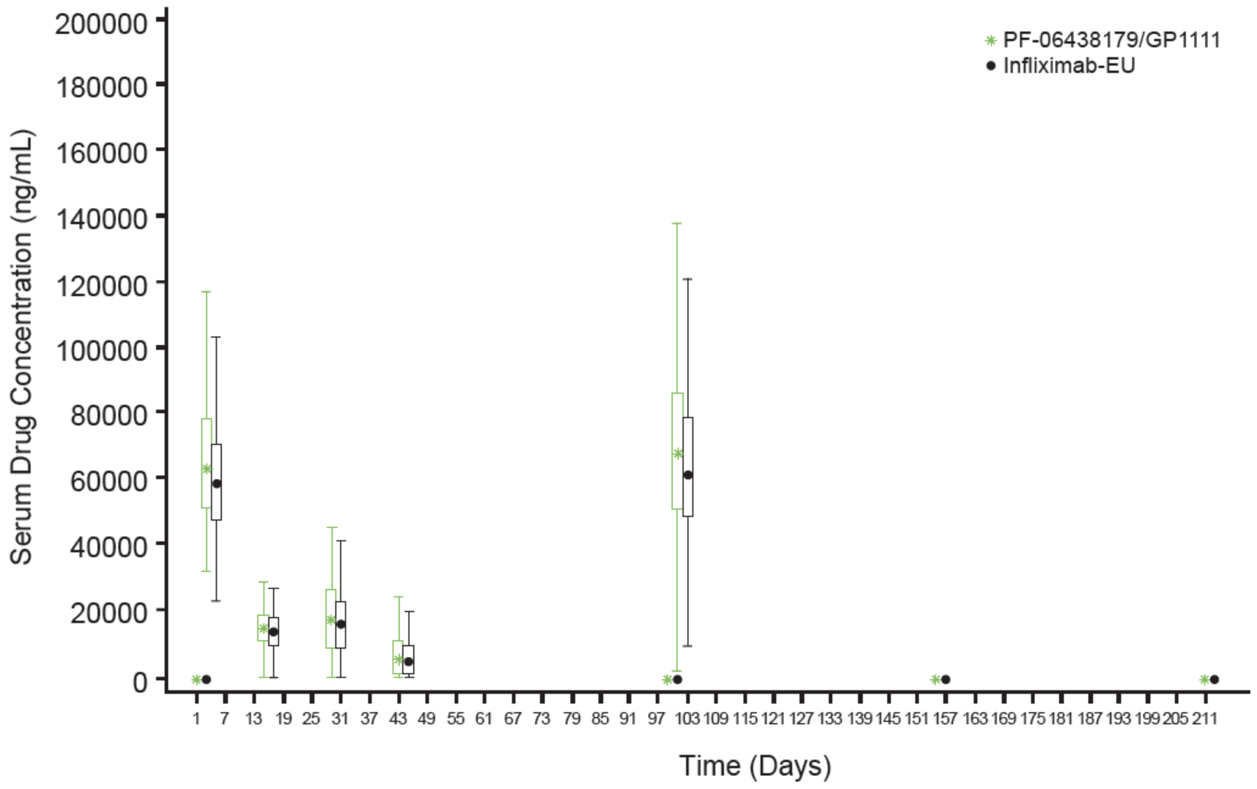

[a] Box plot provides median and 25%/75% quartiles with whiskers to the last point within 1.5x the interquartile range. Both C_{trough} and C_{max} were determined at week 0 (day 1) and week 14 (day 99). Summary statistics were calculated by setting concentration values below the LLOQ (100 ng/mL) to 0.

ADA, anti-drug antibody; C_{max}, observed serum drug concentration prior to the end of infusion; C_{trough}, observed predose trough serum drug concentration; infliximab-EU, infliximab sourced from the European Union; LLOQ, lower limit of quantification; PK, pharmacokinetics.
